# Dominant Gene Expression Profiles Define Adenoid Cystic Carcinoma (ACC) from Different Tissues: Validation of a Gene Signature Classifier for Poor Survival in Salivary Gland ACC

**DOI:** 10.3390/cancers15051390

**Published:** 2023-02-22

**Authors:** Kathryn J. Brayer, Huining Kang, Adel K. El-Naggar, Simon Andreasen, Preben Homøe, Katalin Kiss, Lauge Mikkelsen, Steffen Heegaard, Daniel Pelaez, Acadia Moeyersoms, David T. Tse, Yan Guo, David Y. Lee, Scott A. Ness

**Affiliations:** 1 Department of Internal Medicine, Division of Molecular Medicine, University of New Mexico School of Medicine, Albuquerque, NM 87131, USA; 2 University of New Mexico Comprehensive Cancer Center, Albuquerque, NM 87131, USA; 3 Department of Internal Medicine, Division of Epidemiology, University of New Mexico School of Medicine, Albuquerque, NM 87131, USA; 4 Department of Pathology, University of Texas MD Anderson Cancer Center, Houston, TX 77030, USA; 5 Department of Otorhinolaryngology and Maxillofacial Surgery, Zealand University Hospital, 4600 Køge, Denmark; 6 Department of Pathology, Rigshospitalet, University of Copenhagen, 1165 Copenhagen, Denmark; 7 Department of Ophthalmology, Rigshospitalet-Glostrup, University of Copenhagen, 1165 Copenhagen, Denmark; 8 Dr. Nasser Al-Rashid Orbital Vision Research Center, Department of Ophthalmology, Bascom Palmer Eye Institute, University of Miami Miller School of Medicine, Miami, FL 33136, USA; 9 Sylvester Comprehensive Cancer Center, University of Miami Miller School of Medicine, Miami, FL 33136, USA; 10 Department of Ophthalmology, Bascom Palmer Eye Institute, University of Miami Miller School of Medicine, Miami, FL 33136, USA; 11 The Sheila and David Fuente Graduate Program in Cancer Biology, University of Miami Miller School of Medicine, Miami, FL 33136, USA; 12 Department of Internal Medicine, Division of Hematology/Oncology, Section of Radiation Oncology, University of New Mexico School of Medicine, Albuquerque, NM 87131, USA

**Keywords:** oral cancer, biomarker, MYB oncogene, transcriptome analysis, bioinformatics, survival analysis

## Abstract

**Simple Summary:**

Adenoid cystic carcinoma (ACC) is a pathologically distinctive tumor that most often occurs in major or minor salivary glands, but can also occur in other tissues. We compared the gene expression profiles of ACC tumor samples from salivary gland, lacrimal gland, breast or skin. Despite their different tissues of origin, the ACC tumors displayed highly related patterns of gene expression. Indeed, gene expression patterns could not distinguish ACC tumors from different tissues, suggesting that genetic and epigenetic regulatory events induce a dominant ACC ‘phenotype’. We also used the new cohort of salivary gland ACC tumors to validate a gene expression biomarker developed with a previously analyzed cohort. The 49-gene classifier correctly identified 98% of the poor survival patients, validating the biomarker and suggesting that a clinical test should be developed so patients at highest risk of poor survival can be identified and provided additional treatment.

**Abstract:**

Adenoid cystic carcinoma (ACC) is an aggressive malignancy that most often arises in salivary or lacrimal glands but can also occur in other tissues. We used optimized RNA-sequencing to analyze the transcriptomes of 113 ACC tumor samples from salivary gland, lacrimal gland, breast or skin. ACC tumors from different organs displayed remarkedly similar transcription profiles, and most harbored translocations in the *MYB* or *MYBL1* genes, which encode oncogenic transcription factors that may induce dramatic genetic and epigenetic changes leading to a dominant ‘ACC phenotype’. Further analysis of the 56 salivary gland ACC tumors led to the identification of three distinct groups of patients, based on gene expression profiles, including one group with worse survival. We tested whether this new cohort could be used to validate a biomarker developed previously with a different set of 68 ACC tumor samples. Indeed, a 49-gene classifier developed with the earlier cohort correctly identified 98% of the poor survival patients from the new set, and a 14-gene classifier was almost as accurate. These validated biomarkers form a platform to identify and stratify high-risk ACC patients into clinical trials of targeted therapies for sustained clinical response.

## 1. Introduction

Adenoid cystic carcinoma (ACC) is one of the most common salivary gland malignancies, arising mainly in minor and major salivary glands, but ACC also occurs less frequently in other organs, and the clinical behavior of non-salivary ACC varies widely. This suggests that the ACC tumors arising in different organs may be biologically distinct or that they are affected by different host factors. Molecular analyses have shown that most ACC tumors have recurrent chromosomal translocations that activate the *MYB* oncogene or the related *MYBL1* gene [1,2,3,4], resulting in characteristic gene expression changes [5,6]. The translocations frequently relocate a distant, salivary gland-specific enhancer in proximity to the *MYB* or *MYBL1* genes, leading to their overexpression [7]. Many of the translocations occur within the *MYB* or *MYBL1* genes, leading to truncation and overexpression of the genes and their gene products [5,6]. The *MYB* and *MYBL1* genes encode the DNA-binding transcription factor proteins Myb (c-Myb) and A-Myb, which are important for normal development [8,9]. Relatively small changes in these proteins, such as truncations of the *N*- or *C*-terminal domains, can lead to profound differences in the genes they regulate [10,11,12,13], suggesting that the proteins perform complex regulatory functions. Indeed, the Myb protein can function as a ‘pioneer’ transcription factor capable of initiating the formation of new enhancers that can modify the expression of distant genes [14,15,16]. Thus, Myb or A-Myb proteins activated by *C*-terminal truncations may induce a specific ACC tumor phenotype [17], similar to the actions of Myb proteins in other types of cells and malignancies [18,19,20,21,22,23,24]. Identifying the ACC-specific regulatory mechanisms that induce an ‘ACC phenotype’ could lead to new types of therapies.

ACC patients often have a slow clinical course with a poor long-term prognosis [4,25]. However, clinical outcomes can vary dramatically; unpredictable aggressive and progressive disease is not uncommon. Post-surgical survival ranges from just a few months to 15 years or longer. The protracted temporal progression of ACC tumors necessitates using archived samples at least 5–10 years old for studies linking genomic changes to outcomes. However, standard genomic methods are largely unsuitable for reliable RNA-sequencing (RNA-seq) analysis of archived samples, because the recovered RNA is often highly fragmented, necessitating the use of specialized approaches [5,6,26]. Despite these complications, several studies have identified subgroups of ACC patients with distinct molecular features linked to differences in prognosis and survival [5,6,27,28,29,30], suggesting that applying these approaches to well-structured retrospective cohorts of ACC tumors could produce biomarkers for identifying poor prognosis patients and recommending them for targeted therapy.

In previous studies, we were able to use optimized RNA-seq approaches to successfully analyze the transcriptomes of archived, formalin-fixed, paraffin-embedded ACC tumor samples up to 25 years old, which led to the identification of the first ACC tumors with *MYBL1* translocations [5]. Extending those studies to a larger cohort of 68 samples (the TX cohort) led to the identification of several subgroups of ACC tumors with unique gene expression signatures, including one subgroup with poor survival and a ‘No Myb’ group that expressed neither *MYB* nor *MYBL1* [6]. Although we identified gene expression signatures that correlated with poor survival, it was not possible to validate the results using only one cohort of samples. Here we describe the analysis of a new cohort of ACC tumor samples, from Denmark and Florida (the DK cohort), primarily from salivary gland but also including some ACC tumors from the lacrimal gland, breast, and skin. The availability of the large cohorts allowed us to designate a training set for defining a gene expression classifier to distinguish poor survival samples, which we validated using the second cohort. These results set the stage for using clinical RNA-seq assays for identifying patients who are likely to be in the poor survival subgroup, so they can be offered clinical trials or additional treatment to improve their outcomes.

## 2. Materials and Methods

### 2.1. Human Salivary Gland ACC Samples

De-identified adenoid cystic carcinoma tumor samples were obtained from several institutions: the Department of Otorhinolaryngology and Maxillofacial Surgery, Zealand University Hospital; the Department of Otorhinolaryngology, Head and Neck Surgery and Audiology, Rigshospitalet; the Department of Pathology, Rigshospitalet, University of Copenhagen; and the Department of Ophthalmology, Rigshospitalet-Glostrup, University of Copenhagen, Copenhagen, Denmark. Some lacrimal gland samples were obtained from the Dr. Nasser Al-Rashid Orbital Vision Research Center and the Bascom Palmer Eye Institute, Department of Ophthalmology, University of Miami Miller School of Medicine. All samples were provided Formalin-Fixed and Paraffin-Embedded (FFPE) as 5-micron sections baked onto glass slides. Salivary gland samples with survival information had at least 5-year follow-up. All samples were collected in accordance with the principle of the Declaration of Helsinki and with Institutional Review Board-approved protocols: Danish Regional Ethics Committee (H-6-2014-086) and the Danish Data Protection Agency (Journal no. REG-94-2014).

### 2.2. RNA Isolation and Sequencing

Total RNA was isolated from one or two 5-micron slide-mounted FFPE sections using the RNeasy FFPE kit (Qiagen, 19300 Germantown Rd Germantown MD 20874, USA). cDNA synthesis and library preparation were performed using the SMARTer Universal Low Input RNA Kit for Sequencing (Takara 1290 Terra Bella Avenue Mountain View, CA 94043, USA) and the Ion Plus Fragment Library Kit (ThermoFisher, 168 Third Avenue, Waltham, MA 02451, USA), as previously described [5,6]. Ion Torrent sequencing using Ion S5/XL systems (ThermoFisher) was performed in the Analytical and Translational Genomics Shared Resource at the University of New Mexico Comprehensive Cancer Center. Data are available for download from the NCBI BioProject database using study accession number PRJNA287156. The TX cohort of samples has been described previously [6].

### 2.3. Data Analysis

Low quality and non-human RNA-seq reads were filtered and removed using the Kraken2 suite [31,32,33]. High-quality reads were aligned to the human genome (hg38) using TMAP (v5.10.11), and gene counts were calculated using HT-Seq, as previously described [5,6]. Poor quality samples with fewer than 10% of the median number of reads of all samples were removed. Samples that failed other quality control tests were also removed. The same parameters were used when the data from the new (DK) cohort were combined with the previously described samples in the TX cohort [6]. (Software versions are provided in File S1).

### 2.4. Unsupervised Hierarchical Clustering

For identifying clusters, analyses were limited to genes that were expressed above a threshold level in a number of samples (e.g., 250 reads in at least 10 samples). These thresholds were reduced (e.g., to 50 reads in at least 10 samples) to generate the final heatmaps, to include as many relevant genes as possible, while retaining the clusters and the sample order. Multi-dimensional scaling was performed using plotMDS from the limma package version 3.48.0. Hierarchical clustering was performed using hclust from the stats package (R/Bioconductor version 4.1.0) [34].

### 2.5. Statistical Analysis

Overall survival (OS), defined as time from the date of diagnosis to the date of death, was the primary endpoint for outcome. Subjects who were lost to follow-up or alive within the follow-up period were censored at the date of the last contact. OS was estimated using the Kaplan–Meier method. Differences in OS were examined using the log-rank test. All the statistical analyses were performed using statistical software R (version 4.1.0) [34]. Gene Ontology analyses were performed with Bioconductor package GO.db version 3.13.0, as previously described [5,6,10,26].

### 2.6. Development and Evaluation of Prognostic Classifiers

Personalized logistic regression with the elastic net and LASSO regulations implemented in R package glmnet [35] was used to develop the classifiers that distinguish between the poor prognosis group and the remaining samples. ROC curve and the area under the curve (AUC) were used to evaluate the performance of the classifiers. The prognostic models were built using the training data set, i.e., the TX cohort, through a 10-fold cross-validation (CV) procedure. An unbiased estimate for each of the final models was obtained by performing a nested CV procedure that included the full cycle of the 10-fold inner loop CV followed by a 100 × 6-fold outer loop CV using the training set. The prediction accuracy of each model was further validated using an independent test set, i.e., the DK cohort.

## 3. Results

### 3.1. ACC Tumors from Different Organ Sites Share a Common Transcriptional Profile

Although they most commonly occur in major and minor salivary glands, ACC tumors can also arise in lacrimal, bronchial, mammary, or skin adnexal glands. To assess the similarities and differences in gene expression patterns in ACC tumors from different tissues, we performed RNA-sequencing (RNA-seq) on a cohort of 113 ACC tumors, comprised of 17 samples from breast tissue, 24 from cutaneous tissue, 16 from lacrimal glands, and 56 from salivary glands (Table 1). Most of the samples came from Denmark (DK cohort), with the exception of 6 lacrimal gland samples from Florida (FL). We used optimized methods for RNA analysis and Ion Proton sequencing that we developed previously [5,26]. For the DK cohort, 91 samples (81%) clearly expressed *MYB*, while 13 (12%) expressed *MYBL1* and 9 (8%) expressed neither *MYB* nor *MYBL1*.

**Figure 1:**
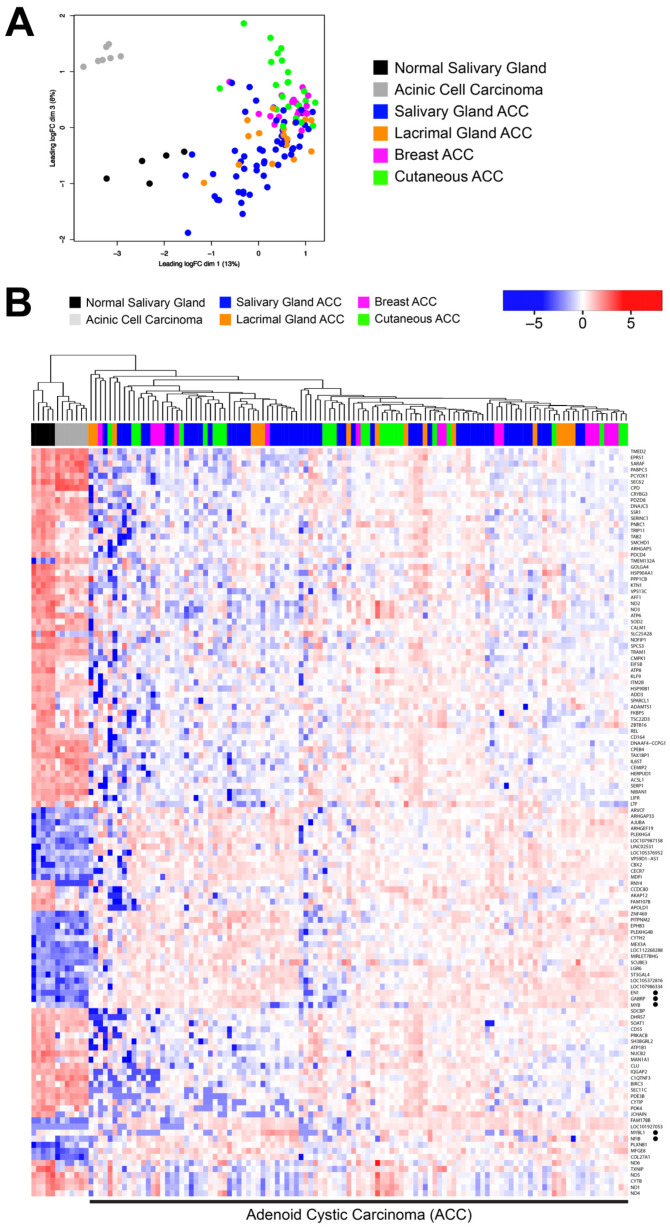
Common features of ACC tumors from different tissues. Differential gene expression analysis was performed on the DK cohort of 113 ACC tumor samples from different tissues, including breast (*n* = 17, pink), cutaneous (*n* = 24, green), lacrimal gland (*n* = 16, orange), and salivary gland (*n* = 56, blue) as well as normal salivary gland (*n* = 5, black) and salivary gland acinic cell carcinoma samples (*n* = 7, gray). (**A**) Multidimensional scaling shows that the acinic cell carcinoma samples clustered at the upper left, the normal salivary gland samples in the lower left, and all of the ACC tumor samples at the right, no matter what tissue they were derived from. (**B**) The heatmap summarizes the gene expression differences. The color bar at the top identifies the tissue type and tissue of origin. The normal salivary gland and acinic cell carcinoma samples are at the left. Several genes important for ACC tumors are marked by dots at the right. (Note: a larger version of this heatmap is provided in Figure S1 in the Supplementary Materials).

We first performed Multi-Dimensional Scaling (MDS, i.e., principal component analysis) for the ACC samples. As shown in Figure 1A, the dots representing ACC tumor samples from different organs are shaded with different colors (see legend). For comparison, we included RNA-seq results from several normal salivary gland tissues (shaded black) and several from a histologically different salivary gland tumor, acinic cell carcinoma (shaded gray), that have been described previously [5,28]. All the ACC tumor samples clustered into a large group at the right, suggesting that the transcription profiles of the ACC tumors are more similar to each other than they are to normal tissue or other salivary gland tumors, despite the different tissues of origin.

Next, we used unsupervised hierarchical clustering to group samples that were similar and generated the heatmap comparing the transcriptional profiles shown in Figure 1B. Interestingly, as shown by the dendrogram at the top of the heatmap, the ACC tumor samples formed several large subgroups, but each cluster contained samples from all the tissue types: salivary gland, lacrimal gland, breast and cutaneous (dark blue, orange, pink and green in the color bar at top, respectively). Thus, although the ACC samples in this cohort are heterogeneous and formed distinct subgroups, the groups are not defined by the tissue of origin. Instead, the subgroups could represent biological differences amongst the ACC samples, irrespective of the tissue from which they were derived. These results differ somewhat from a previous report showing that microRNA expression profiles could distinguish ACC tumors from different tissue types [36], suggesting that the biological mechanisms leading to the formation of ACC tumors may have a more profound impact on the overall mRNA transcriptional profile than on microRNAs, which may be more tissue-specific. Several genes that are known to be important in ACC tumors are highlighted with black dots at right, including (from top to bottom) *EN1*, *GABRP*, *MYB*, *MYBL1* and *NFIB*, all of which are expressed more highly in the ACC tumors than in the normal salivary gland or acinic cell carcinoma samples (at left).

### 3.2. Many ACC Tumors Fail to Express Tissue-Specific Gene Expression Markers

**Figure 2:**
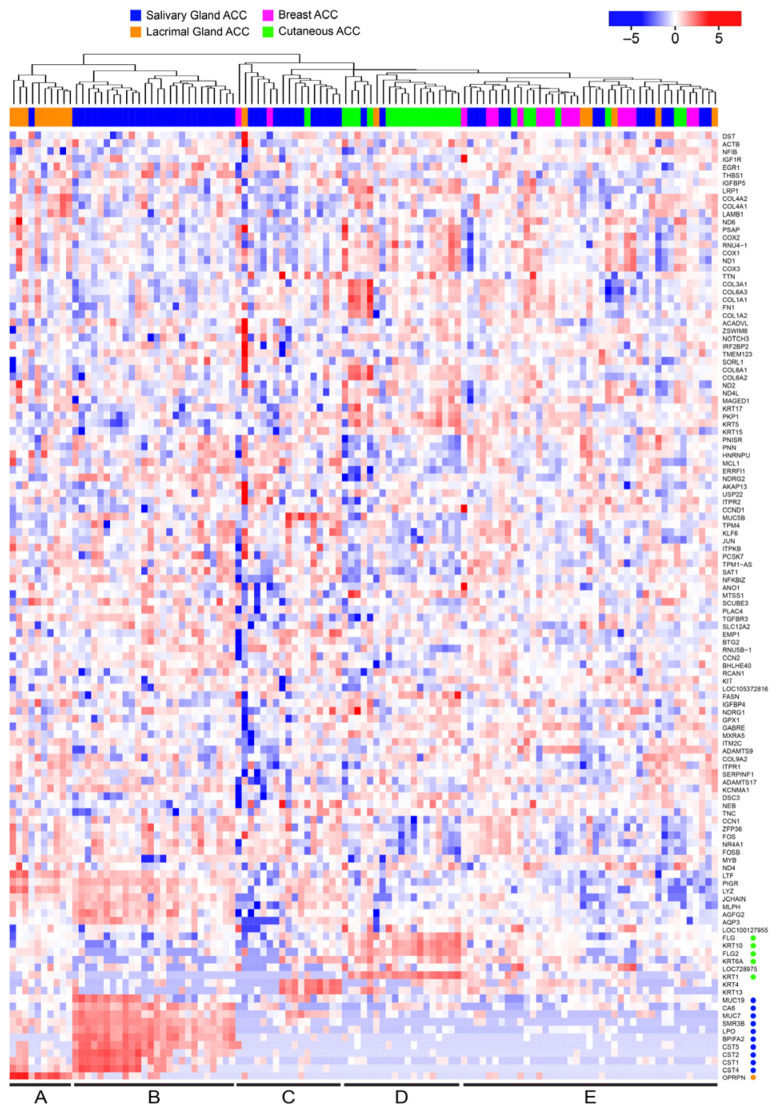
Tissue-specific gene expression differences in ACC tumors. The ACC tumors from the DK cohort were analyzed for tissue-specific gene expression by specifically selecting genes that marked tumors derived from different tissues. A total of 1089 differentially expressed genes were identified by comparing all the tissue groups to each other (at least 2-fold up- or down-regulated with adjusted *p*-value < 0.05). The heatmap summarizes the gene expression differences for 123 of the most highly expressed genes. The tissues of origin are indicated in the color bar at top: lacrimal gland, salivary gland, cutaneous, and breast are indicated by orange, blue, green and pink, respectively. Notable genes mentioned in the text are marked by dots along the right edge. (Note: a larger version of this heatmap is provided in Figure S2 in the Supplementary Materials).

Since our initial hierarchical clustering did not separate ACC tumors by tissue type, we reanalyzed the data to see if we could identify tissue-specific gene expression patterns in the tumors. We specifically selected genes that were differentially expressed in the ACC tumors originating in different tissues. The heatmap in Figure 2 summarizes the results when the most tissue-specific genes are chosen for display (and the normal salivary gland and acinic cell carcinoma samples are left out). This type of analysis led to better clustering of the ACC samples from lacrimal gland (orange, left), salivary gland (blue), cutaneous (green) and breast (pink). There were specific genes that were up-regulated in some ACC samples compared to the others. For example, at the far left of the heatmap (labeled A at bottom, orange color bar at top) is a group of ACC tumors, mostly from the lacrimal gland, that overexpress the *OPRPN* gene, which encodes the Opiorphin Proline-Rich Lacrimal Protein 1 (previously named *PROL1*). The next group (labeled B) are salivary ACC (blue color bar at top) and overexpress several salivary-gland specific genes including *MUC19*, *CA6*, *MUC7*, *SMR3B*, *LPO*, *BPIFA2*, *CST5*, *CST2*, *CST1* and *CST4* (marked by blue dots at lower right of figure). Group C also contains salivary ACC samples with a few from other tissues, and several overexpress *KRT4* and *KRT13*. Most of the cutaneous samples clustered in a group (D, green color bar) and are identified by overexpression of *FLG*, *KRT10*, *FLG2*, *KRT6A* and *KRT1*. However, the remaining ACC tumors formed a large cluster (labeled E), which included most of the breast ACC tumors as well as samples from salivary, lacrimal and cutaneous adnexal glands. The last group was notable because the samples failed to express the gland-specific marker genes that defined the other groups, suggesting that they had a more de-differentiated or perhaps more stem cell-like phenotype. Thus, while we were able to identify tissue-specific marker genes in some ACC tumors, the specificity was not absolute and there remained significant heterogeneity in the gene expression patterns of different samples. Also, since tumor samples always contain some normal cells, the tissue-specific differences that were detected could be due to the non-tumor cells in the samples. We conclude that an ACC-specific gene expression pattern dominated the tumors, apparently overriding the tissue-specific differences.

### 3.3. The New DK Cohort of ACC Tumors Also Contains a Poor-Survival Subgroup of Patients

**Figure 3:**
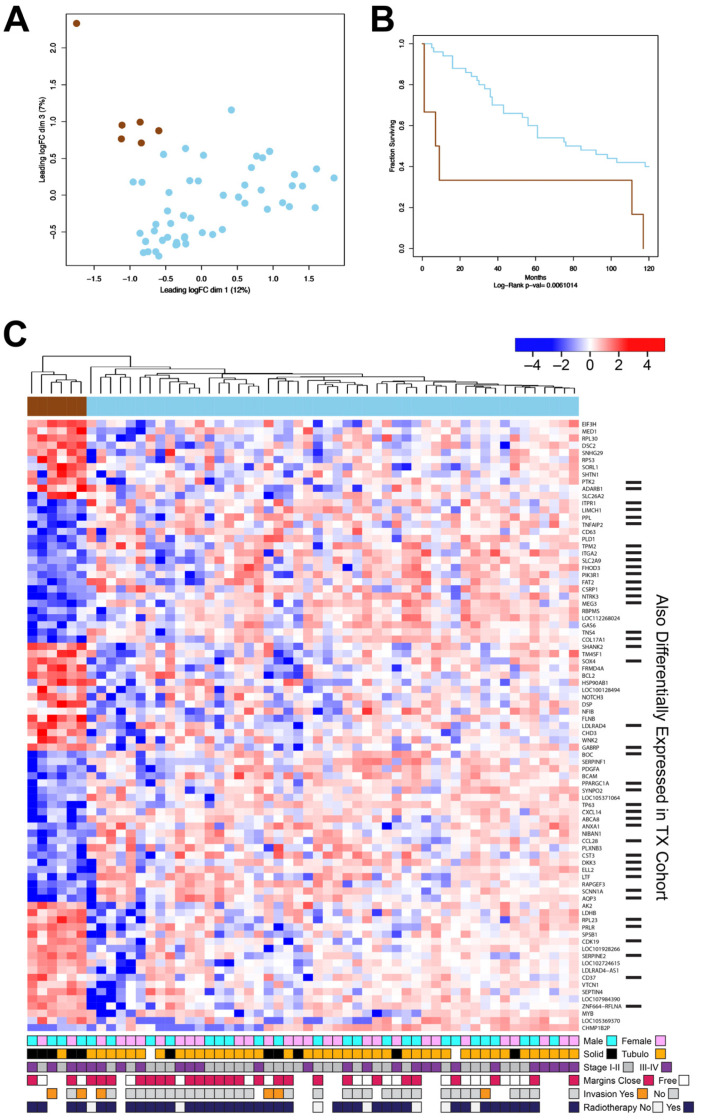
The DK cohort of ACC tumors contains a poor-outcome subgroup. (**A**) Multi-dimensional scaling plot of the gene expression data from the DK cohort of salivary gland ACC tumor samples. (**B**) Kaplan–Meier survival analysis shows that the samples in the brown subgroup had a significantly (*p*-value = 0.006) worse survival. (**C**) The heatmap summarizes the differential gene expression analysis comparing the poor survival (brown) subgroup to the rest of the samples. Genes marked by bars at the right were also identified previously in a poor-survival subgroup from the TX cohort [6]. The color bars at the bottom summarize the available clinical information for gender, solid or tubulocribiform morphology, tumor stage, margins, vascular invasion, and radiotherapy. A larger version of the heatmap is provided in Supplementary Figure S3.

Previous studies of ACC tumor samples identified subgroups of tumors with distinct gene expression and survival characteristics [6]. We carefully evaluated the new cohort of salivary gland ACC tumors for evidence of subgroups with distinct gene expression patterns. Figure 3A shows a multi-dimensional scaling plot of the 56 DK cohort salivary gland samples. Most of the tumors (shaded light blue) form a large cluster but a small group of tumor samples (brown) formed a separate group at the upper left corner of the plot. Figure 3B shows a Kaplan–Meier survival analysis: the samples in the brown group had a median survival of only 8 months, compared to the main group (light blue), which showed a median survival of 80 months (*p*-value = 0.006). This is reminiscent of our previous results with the TX cohort, where a subgroup of ACC tumors displayed similarly poor survival [6]. Some ACC tumors display a ‘solid form’ morphology, which has been associated with worse prognosis [37,38]. Although ‘solid morphology’ ACC tumors were excluded from the TX cohort, the DK cohort contains 11 such samples: 5 in the poor prognosis group and 6 in other group. This suggests that the poor prognosis group is not defined simply by solid tumor morphology.

The differential gene expression analysis identified 273 genes at least 2-fold up- or down-regulated in the brown subgroup (adjusted *p*-value < 0.05). The results for 85 of the genes are summarized in the heatmap in Figure 3C (The poor-survival subgroup cluster is at the left side of the heatmap, marked by the brown color bar at the top). In the heatmap, all the genes that were regulated in similar directions both in this new DK cohort and also in the previously described TX cohort (e.g., up-regulated in both poor survival groups) are marked by bars along the right edge of the figure. Genes that were up-regulated in both poor-survival subgroups include (from bottom of the heatmap) *CD37*, *SERPINE2*, *CDK19*, *PRLR*, and *RPL23*. Down-regulated genes include *AQP3*, *SCNN1A*, *LTF*, *ELL2*, and *DKK3*. These results further indicate that a subgroup of ACC tumors from patients with poor survival display a unique gene expression profile that could potentially be used for prognostication [6].

### 3.4. Combining the TX and DK Cohorts Provides Additional Details about Subgroups of ACC Tumors

**Figure 4:**
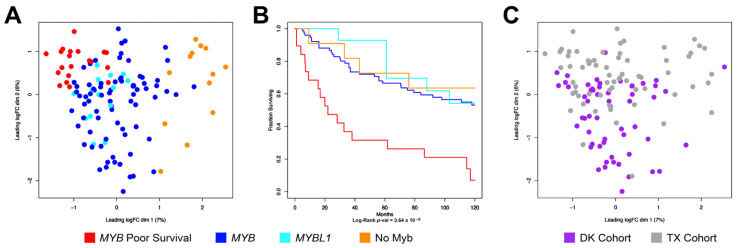
Combined analysis of the DK and TX cohorts of ACC tumor samples. The RNA-seq data from the new DK cohort was combined with previously described (Frerich et al., 2018 [6]) data from the TX cohort for a combined gene expression analysis. (**A**) Combined multi-dimensional scaling plot. Orange shading indicates ‘No Myb’ samples that express neither *MYB* nor *MYBL1*, blue and cyan indicate *MYB*- or *MYBL1*-expressing samples in the main group, red indicates the *MYB*-expressing samples with poor survival. (**B**) Kaplan–Meier survival plot of samples in the four groups of ACC samples. (**C**) Multi-dimensional scaling plot similar to panel A, but with samples from DK or TX cohorts labeled purple or gray, respectively.

The results described above suggest that the new DK cohort of ACC tumor samples contains subgroups of patients that are very similar to the subgroups we identified previously in the TX cohort [6]. To compare the subgroups we combined the RNA-seq results of the two independent cohorts and performed a unified analysis of 124 salivary gland ACC samples (56 from DK and 68 from TX). Figure 4A shows the multi-dimensional scaling plot of the combined data sets, which form three main groups. The largest group, in the middle of the plot, have been shaded dark blue or cyan to indicate that they overexpress *MYB* or the related *MYBL1* gene, respectively. A group at the upper left is shaded red and contains the poor survival samples from both cohorts, all of which express MYB. Finally, a group of samples at the right, shaded orange, express neither *MYB* nor *MYBL1* (‘no *MYB*’). This group was described previously, and the ‘driver’ oncogenes or mutations responsible for that group remain unknown [6]. Although these cohorts of ACC samples were completely independent and the patients came from different countries, both cohorts formed similar major subgroups when analyzed together. A Kaplan–Meier survival analysis of these groups is shown in Figure 4B. The overall survival for patients in the orange ‘no Myb’ group was similar to the main group of samples expressing either *MYB* or *MYBL1*. As described above, the red group displayed much worse survival compared to the other patients. While the median survival for most patients exceeded 120 months, including the orange ‘no *MYB’* group, median survival for the red group was only 16.8 months (*p*-value < 1 × 10^−6^). These groups were segregated using only their different gene expression characteristics, suggesting that biomarkers could be developed to identify the patients in the poor survival group at the time of surgery. The MDS plot in Figure 4C shows the large overlap in the DK and TX cohorts, despite being analyzed separately and several years apart.

### 3.5. ACC Tumors That Do Not Express MYB or MYBL1 Have a Unique Transcription Profile

**Figure 5:**
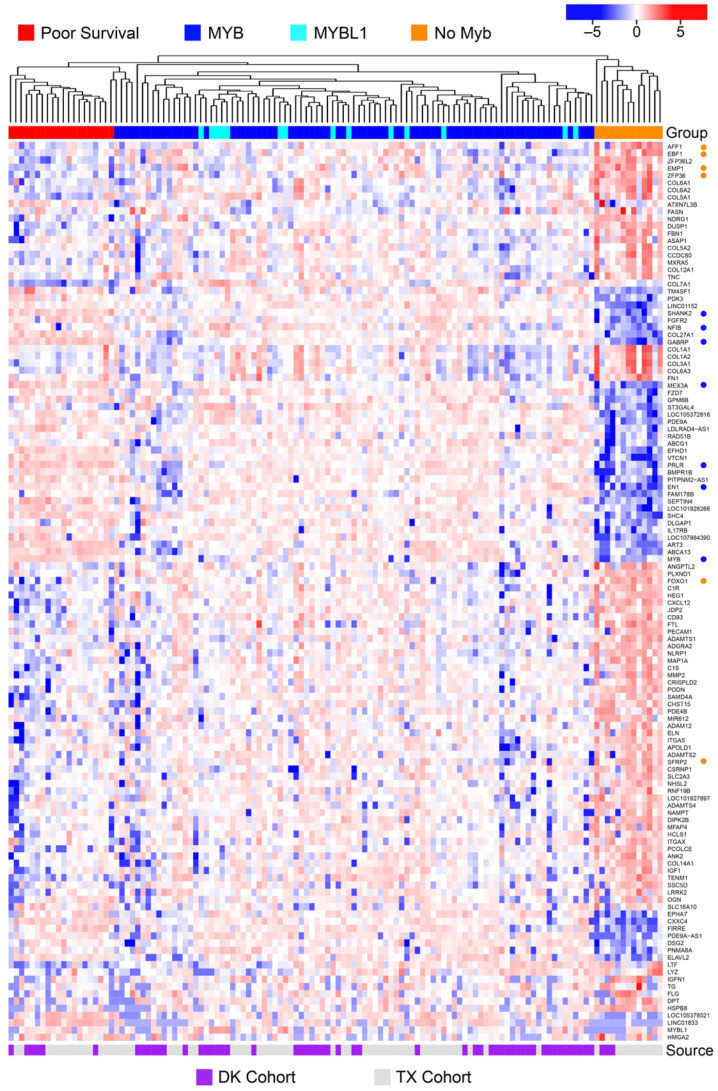
Differential gene expression analysis: ‘No Myb’ samples. The heatmap summarizes the differential gene expression analysis using the combined cohorts of ACC samples from DK and TX, comparing the ‘no Myb’ group (orange color bar at top) to the rest of the samples, all of which express either MYB or MYBL1. Notable genes mentioned in the text are marked by dots at the right. The purple and white color bar at the bottom indicates samples from the DK and TX cohorts, respectively. A larger version of this heatmap is provided in the Supplementary Materials, as Figure S4.

Most ACC tumors have recurrent chromosomal translocations that activate the *MYB* oncogene or the related *MYBL1* gene [1,2,3,4], but this raises questions about the underlying biology and driver genes active in the remaining ACC tumors that do not express *MYB* or *MYBL1*. As shown in Figure 4B, the ‘no MYB’ subgroup of tumors (orange line) had survival similar to the bulk of ACC samples (blue and cyan lines). To further explore the potential driver genes in these samples, we compared them to the rest of the ACC samples in the combined cohort and performed a differential gene expression analysis. In the heatmap shown in Figure 5, the dendrogram at the top shows the hierarchical clustering that was used to arrange the samples from left to right. The ‘no *MYB*’ samples are at the far right (marked by orange at the top). The heatmap summarizes the gene expression differences for 124 of the 881 genes that were differentially expressed (at least 2-fold up- or down-regulated, adjusted *p*-value < 0.05) when the ‘no *MYB*’ samples were compared to all the others. The top 10 up- or down-regulated genes are listed in Table 2, and the full list is provided in Supplementary Data (Table S1).

There are several important conclusions from this analysis. First, as described previously [5,6], the ACC samples that express *MYBL1* do not form their own subgroup, but mix in with the samples expressing *MYB*, suggesting that the two oncogenes have similar effects on gene expression patterns [6]. Second, each of the three main groups (orange, red, blue) contains samples from both the TX and DK cohorts, suggesting that these subgroups are consistent in ACC tumors and are not a characteristic unique to just one cohort or one analysis. Several interesting genes are marked along the right side of the heatmap, including *AFF1*, *EBF1*, *EMP1*, *ZFP36*, *FOXO1*, and *SFRP2*, which are all up-regulated in the ‘no *MYB*’ tumors (marked by orange dots). The *SHANK2*, *NFIB*, *GABRP*, *MEX3A*, *PRLR*, and *MYB* genes were down-regulated in the ‘no *MYB*’ tumors (marked by blue dots). A gene set enrichment analysis identified a number of Gene Ontology Cellular Process categories that were over-represented in the differentially expressed genes. The top six categories are described in Table 3. The finding that the ‘no MYB’ samples have such a dramatically different gene expression profile reinforces the conclusion that the ACC phenotype can be achieved through different regulatory pathways.

### 3.6. Poor Survival ACC Samples Have a Unique Transcription Profile

For the combined cohorts, the poor survival subgroup, marked by red at the top of the heatmap in Figure 5 and in the survival plot in Figure 4B, displayed a median survival of only 22 months, compared to greater than 123 months for the other patients. This suggests that a gene expression panel could be developed to identify patients at highest risk of poor survival.

**Figure 6:**
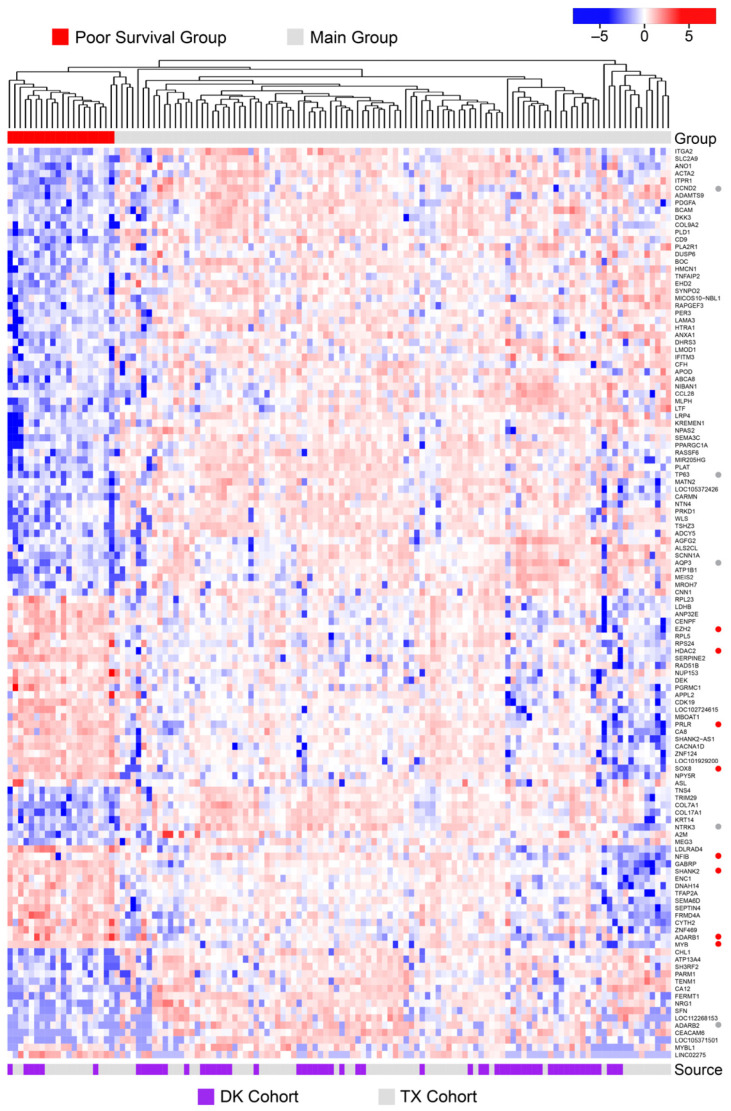
Differential gene expression analysis: Poor survival samples. The heatmap summarizes the differential gene expression analysis using the combined cohorts of ACC samples from DK and TX, comparing the poor survival group (red color bar at the top) to the rest of the samples. Notable genes mentioned in the text are marked by dots at the right. Red and blue dots indicate genes up- or down-regulated in the poor survival samples. The purple and white color bar at the bottom indicates samples from the DK and TX cohorts, respectively. A larger version of this heatmap is provided in the Supplementary Materials, as Figure S5.

To characterize the poor survival subgroup in more detail, we performed in depth analysis of the gene expression patterns of tumors from these patients. The heatmap in Figure 6 summarizes the results of a differential gene expression analysis comparing the poor survival subgroup samples to all the other ACC tumor samples in the combined cohort. The samples are arranged in the same left-to-right order as in Figure 5, using the dendrogram generated by hierarchical clustering, and the poor-survival samples are indicated by the red color bar at the top. The samples from the TX and DK cohorts are indicated by the gray and purple color bar at the bottom, respectively. The heatmap summarizes the relative expression of the 124 most differentially expressed genes out of the 729 genes that were at least 2-fold up- or down-regulated (with adjusted *p*-values > 0.05). Several notable up- or down-regulated genes are marked by red or gray dots, respectively, along the right side of the heatmap. The genes that were up-regulated in the poor survival samples include *EZH2*, *HDAC2*, *PRLR*, *SOX8*, *NFIB*, *SHANK2*, and *ADARB1*. The down-regulated genes include *CND2*, *TP63*, *AQP3*, *NTRK3*, and *ADARB2*. The top 10 up- or down-regulated genes are listed in Table 4 and the full list is provided in Supplementary Data (Table S2). Interestingly, there is no single gene that is specifically up- or down-regulated only in the poor survival samples, or that could be used to identify either the poor survival or better survival patients, suggesting that a multi-gene biomarker could be developed to identify the patients in the poor-survival subgroup.

### 3.7. A Multi-Gene Classifier to Identify Poor Survival Patients

As shown in Figure 3, the DK cohort of ACC samples contained a subgroup of patients with poor survival, similar to one that was originally identified in the TX cohort [5,6]. Having patients from two independent cohorts allowed us to use the original TX cohort as a training set to develop a multi-gene biomarker panel, which could be validated with the DK cohort. Starting with expression data for 3597 genes expressed above a threshold level in the training set (TX cohort), we used an elastic net type of penalized logistic regression model to identify genes that could distinguish the poor prognosis cohort from the rest of the patients. The model selection was performed with a 10-fold cross-validation and yielded a 49-gene classifier developed solely with data from the TX cohort (Table 5). The ROC curve analysis was used to evaluate the classifier’s accuracy.

**Figure 7:**
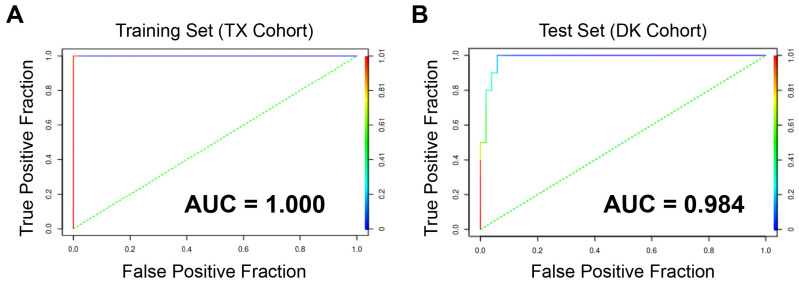
Elastic net ROC curves. The elastic net classifier developed with the TX cohort training set (**A**, **Left**) produced an Area Under the Curve (AUC) of 0.984 in the DK cohort test set (**B**, **Right**).

As shown in Figure 7, the elastic net classifier could distinctly separate the poor prognosis samples from others in the training set (TX cohort, left panel) as AUC = 1. An unbiased estimate for the AUC (AUC = 1) was also achieved through a double loop nested cross-validation, which showed a perfect classification performance. However, the accuracy achieved was expected because the same gene expression data were used to develop and test the classifier. Importantly, the classifier developed with the TX cohort also gave nearly perfect (AUC = 0.984) separation on the independent DK cohort test set (right panel). We also generated a 14-gene subset of the classifier using a Least Absolute Shrinkage Selection Operator (LASSO) approach. The 14-gene classifier containing genes *A2M*, *ACTA2*, *ANO1*, *APOL6*, *DMD*, *IPO9*, *LIMCH1*, *MAMLD1*, *MIR205HG*, *PLAT*, *RASSF6*, *SEMA3C*, *SLPI*, and *TP63* (shown in bold in Table 4) was only slightly less accurate, with AUC = 0.976. These results suggest that a gene classifier can be used to identify ACC patients with poor prognosis.

**Figure 8:**
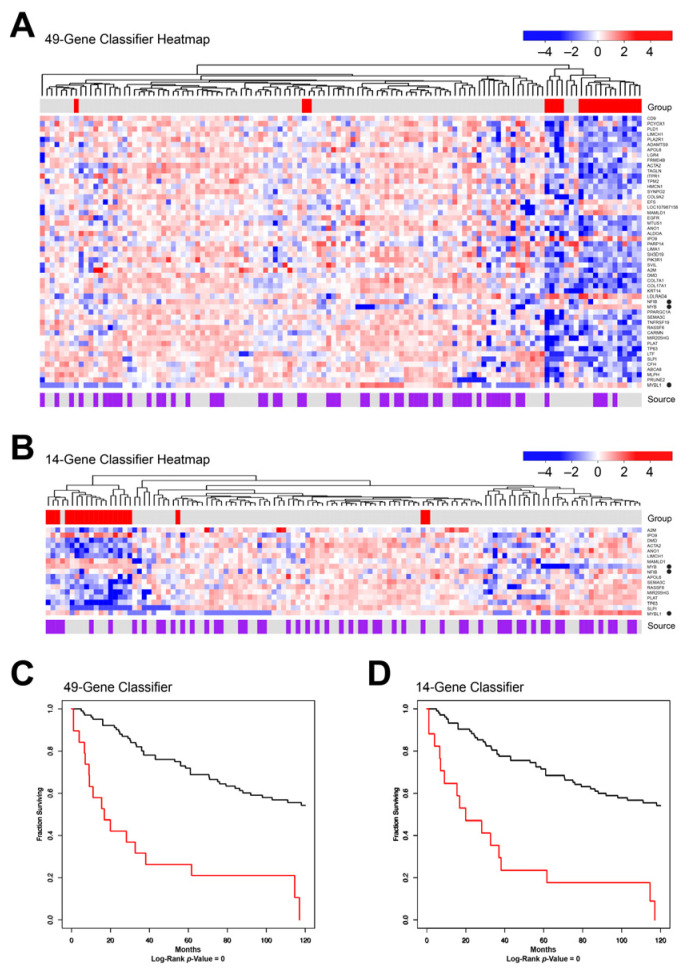
Classifier Groups. The 49-gene (**A**) or 14-gene (**B**) classifiers were used to separate ACC samples into groups by hierarchical clustering, as illustrated in the dendrograms at the top of each heatmap, which compare the gene expression profiles of the two groups. (The *MYB*, *MYBL1*, and *NFIB* genes were added to the analysis for comparison. They are marked by black dots at the right). The samples from the original poor survival group (see Figure 4) are marked by red in the color bars. At the bottom, Kaplan–Meier plots compare the survival of patients in the groups defined by the 49-gene (**C**) or 14-gene (**D**) classifiers. Larger versions of these heatmaps are in Supplementary Figure S6.

To illustrate their usefulness, we tested the genes in the classifiers on the combined DK and TX cohorts of salivary gland ACC samples. We limited the data sets to only the 49-gene or 14-gene lists (except that *MYB*, *MYBL1*, and *NFIB* were added back for comparison), and performed hierarchical clustering, which identified the two major clusters shown in the dendrograms in Figure 8A,B. The heatmaps display the differences in gene expression. Interestingly, most of the classifier genes were down-regulated in the poor prognosis tumors compared to the other samples. As an example, the *TP63* tumor suppressor gene is significantly down-regulated in the poor prognosis group. The poor prognosis tumors appear to lack the expression of specific genes that are expressed by the other ACC samples. Notably, only 4 of the 11 ‘solid form’ morphology samples from the DK cohort were in the poor prognosis subgroup, suggesting that solid morphology is insufficient to classify samples as poor prognosis [37,38].

As shown by the color bar at the top of each heatmap, some of the samples that were in the poor prognosis subgroup described in Figure 4 (marked red) did not cluster with the poor survival samples identified by the gene classifiers, and a few samples that were not included above did cluster in the poor survival group in this analysis. However, as shown in the Kaplan–Meier survival plots in Figure 8C,D, the classifiers did identify a poor-prognosis group with median survival of less than 20 months, compared to a median of 125 months for the rest of the samples. In addition, none of the poor-prognosis patients identified by the classifiers survived 10 years, while more than half of the other patients survived at least 10 years. Thus, the multi-gene classifiers identified using the TX cohort samples were able to identify a subset of ACC patients in the independent DK cohort, which validates the classifiers and suggests that adapting them to the clinic could be useful.

To examine whether the gene classifier provides more information for survival outcomes beyond that contained in the clinical covariates, we performed univariate and multivariate Cox regression analyses, with the gene classifier and clinical covariables deemed to be the risk factors as predictors. (Details of the analysis are in File S1). The available clinical covariables include Margins (free or close), Vascular Invasion (yes or no), Radiotherapy (yes or no), Cribriform (tubular or solid), and Stage (I-II or III-IV). The analyses were restricted to 56 samples; a union of the subsets to which the data of each variable are available. However, the number of samples used by each Cox regression analysis varied subject to data availability. The univariate and bivariate analyses are in the Supplementary Materials, and the multivariate analysis is reported in Table 6.

The univariate analysis (see Supplementary Materials) showed that the two variables, Vascular Invasion and Cribriform, were significantly associated with survival outcomes (*p* < 0.05), while the variable Stage was marginally significant (*p* = 0.084). We compared these three variables with the gene classifier through bivariate Cox regression (see Supplementary Materials). The result showed a remarkable association between our gene classifier and survival after adjusting for each clinical covariate’s effect. We further performed a multivariate Cox regression (Table 6), and our gene classifier was still significantly correlated with the survival outcomes after adjusting for Vascular Invasion and Stage effects. Note that the Cox regression with three or more variables will not converge if we include Cribriform in the model, which limits our ability to conduct further investigation in this respect. However, the results have given sufficient statistical evidence that our gene classifier provided more information about the survival outcome than the available clinical parameters.

## 4. Discussion

We compared the transcription profiles of ACC tumor samples that arose in very different tissues: salivary gland, lacrimal gland, breast, and skin. Despite being from different tissues, all ACC tumors had markedly similar gene expression profiles. Indeed, the ACC samples were much more similar to each other than they were to normal salivary gland tissue or another type of salivary gland tumor, acinic cell carcinoma [28]. These results demonstrate that ACC tumors arising in different tissues are highly related and are difficult to distinguish using gene expression patterns alone. Interestingly, different types of ACC tumors were shown previously to have distinct patterns of microRNA expression [36]—a result that we could not reproduce using gene expression results. This suggests that the activated *MYB* or *MYBL1* oncogenes may induce an ACC-specific gene expression pattern that affects protein-coding genes much more than microRNAs. This is a fascinating biological difference that could be important for explaining tumor phenotypes and some aspects of tissue differentiation.

Having RNA-seq data from a new set of ACC samples provided us with the opportunity to perform a validation cohort analysis. Despite the challenges that exist for translating RNA sequencing (RNA-seq) results into widely used clinical assays [39], several types of gene expression signatures have been developed for clinical use [40,41,42]. In this study, we used RNA-seq data from a previous cohort of 68 salivary gland ACC samples to develop a 49-gene expression classifier for identifying a subgroup of patients with poor survival. We then validated the result using results from the new cohort of 56 salivary ACC samples, finding that the biomarker was able to distinguish 98% of the poor survival patients. A smaller 14-gene classifier achieved similar results with slightly less accuracy. Salivary gland ACC patients display widely variable outcomes, with some patients surviving decades after surgery and others succumbing after only a few months [5,30,43]. It seems clear that the development of new clinical trials should be targeted to the ACC patients that are most likely to have a recurrence and die from the disease. The validated biomarker we have described should have important utility, if it can be developed into an assay suitable for clinical laboratory use. Although our results do not suggest a new or modified treatment for ACC patients, they do suggest that developing a suitable biomarker assay to identify the worse prognosis patients is worthwhile so a new therapeutic strategy could be developed for them. Clinical RNA-seq is fast becoming a routine assay for cancer patients, so these biomarkers should be adaptable to clinical laboratories.

Some ACC tumors display a ‘solid form’ morphology, which has been associated with worse prognosis [37,38]. Other clinical features, such as advanced tumor stage, lack of clean margins during surgery, or vascular invasion, might also be used to identify higher risk patients. However, in our analysis, none of these other markers were able to identify the poor prognosis group of patients that we identified using gene expression patterns. Therefore, we conclude that the gene classifiers provide a novel and independent means of distinguishing poor prognosis ACC patients that should be pursued and studied further. The next step will be to develop assays that work in clinical laboratories so that these classifiers can be used to identify patients that should be targeted for clinical trials or more aggressive therapy to improve their survival.

In addition to identifying and validating a multi-gene classifier for ACC patients, analyzing a new cohort of 56 salivary gland ACC samples from Denmark (DK) also validated important biological results that we described previously using 68 ACC patient samples from the Salivary Gland Tumor Bank in Texas (TX) [5,6]. The main result is that ACC patients can be divided into at least three distinct groups based on gene expression signatures. These groups are easily discernable in the multi-dimensional scaling plots (e.g., see Figure 4). The samples in the main group, comprising 76% of the total, express either *MYB* or *MYBL1* and have a median survival of more than 10 years after surgery. A second group, about 10% of the samples with survival similar to the main group, express neither *MYB* nor *MYBL1*. These samples have a unique gene expression signature, suggesting a different mechanism driving the malignancy. The samples in the final group, about 14% of the total, are the focus of the multi-gene classifier because they have much worse survival than the rest of the ACC patients.

Although the detailed transcriptome analyses that we performed were able to discern distinct subgroups of ACC tumor types, the bulk RNA-sequencing does not provide information on cell lineage composition within tumors. Thus, it is not clear if the different subgroups result from the unique features of different types of ACC tumor cells or whether the subgroups are due to differences in cellular composition in the tumors. Addressing those questions will require using single-cell genomics assays or spatial genomics approaches that can discern different cell types in the tumors.

## 5. Conclusions

Our somewhat surprising result is that ACC tumors arising in different tissues or organs have remarkably similar transcriptional profiles. Indeed, we were unable to identify gene signatures that distinguished the ACC tumors from different organs. This may point to an important underlying biology in ACC tumors that makes them so similar. Since the majority of ACC tumors overexpress the *MYB* or *MYBL1* genes, the dominant ACC phenotype may be induced by the activated Myb transcription factors.

A second, but very important finding is that RNA sequencing analysis can be used to identify a subgroup of MYB-expressing salivary gland ACC patients with poor prognosis. We were able to use the new DK cohort of ACC samples to validate a biomarker developed with an earlier (TX) cohort. This is especially important for diseases like ACC, in which many patients survive more than 10 years post-surgery. Our results provide a tool for identifying the patients that should be enrolled in clinical trials of targeted therapies to improve their outcomes.

## Figures and Tables

**Table 1:** ACC Tumor Cohorts.

Tissue	Number	Form	Gender	Stage	Age at Surgery	Source	Oncogene *
New Cohort (DK)							
Breast	17	NA	NA	NA	NA	DK	*MYB*: 16 *MYBL1*: 1
Cutaneous	24	NA	NA	NA	NA	DK	*MYB*: 17 *MYBL1*: 3 No_*MYB*: 4
Lacrimal	16	NA	NA	NA	NA	DK: 10 FL: 6	*MYB*: 11 *MYBL1*: 2 No_*MYB*: 3
Salivary	56	Solid: 11 Tubulocribiform: 43 NA: 2	F: 29 M: 27	Stage I-II: 30 Stage III-IV: 26	Age: 32–87 NA: 17	DK	*MYB*: 47 *MYBL1*: 7 No_*MYB*: 2
Cohort Total	113						*MYB*: 91 *MYBL1*: 13 No_*MYB*: 9
Previous Cohort (TX, Frerich et al. 2018 [6])							
Salivary	68	NA	F: 30 M: 38	NA	NA	TX	*MYB*: 52 *MYBL1*: 7 No_*MYB*: 9
Combined	181						*MYB*: 143 *MYBL1*: 20 No_*MYB*: 18

* No *MYB* indicates no expression of either *MYB* or *MYBL1*.

**Table 2:** Differentially Expressed Genes in No MYB Samples vs. All Others.

DE Genes	Top 10 Up-Regulated	FC	Adj *p*-Value	Top 10 Down-Regulated	FC	Adj *p*-Value
881	*TG*	136.26	1.41 × 10^−32^	*LINC01833*	0.02	1.39 × 10^−5^
	*HMGA2*	42.11	1.66 × 10^−14^	*MUC7*	0.03	1.25 × 10^−2^
	*IGFN1*	14.91	7.41 × 10^−8^	*LOC643201*	0.03	2.04 × 10^−3^
	*LYZ*	12.38	4.19 × 10^−6^	*MUC19*	0.03	1.93 × 10^−3^
	*FLG2*	12.08	5.87 × 10^−5^	*CTNND2*	0.04	6.12 × 10^−5^
	*COL1A1*	9.62	9.72 × 10^−20^	*FIRRE*	0.04	4.73 × 10^−10^
	*FLG*	8.80	5.38 × 10^−6^	*LOC105378521*	0.04	8.12 × 10^−6^
	*LTF*	8.05	1.37 × 10^−6^	*ART3*	0.04	7.41 × 10^−8^
	*HSPB8*	7.97	5.59 × 10^−6^	*LOC107984390*	0.04	1.42 × 10^−11^
	*TNNT1*	7.74	2.29 × 10^−3^	*SIX3*	0.04	5.12 × 10^−4^

**Table 3:** Gene Ontology Categories of DE Genes from No *MYB* Group Analysis.

GO.ID	Term	Annotated	Signif	Expected	Adj *p*-Value
GO:0043062	extracellular structure organization	218	87	31.6	2.40 × 10^−19^
GO:0045229	external encapsulating structure organization	219	87	31.74	3.50 × 10^−19^
GO:0045765	regulation of angiogenesis	128	47	18.55	2.40 × 10^−10^
GO:0022610	biological adhesion	658	166	95.37	3.10 × 10^−10^
GO:0008284	positive regulation of cell population prolif.	354	93	51.31	7.80 × 10^−8^
GO:0009617	response to bacterium	178	54	25.8	2.10 × 10^−7^

**Table 4:** Poor Survival Samples vs. All Others.

DE Genes	Top 10 Up-Regulated	FC	Adj *p*-Value	Top 10 Down-Regulated	FC	Adj *p*-Value
729	*ANKRD1*	10.28	3.99 × 10^−5^	*CST4*	2.2 × 10^−04^	3.46 × 10^−3^
	*LINC02275*	8.57	2.77 × 10^−5^	*CST5*	3.0 × 10^−03^	3.82 × 10^−3^
	*LINC02515*	5.66	4.90 × 10^−5^	*CST2*	3.0 × 10^−03^	8.83 × 10^−3^
	*NCAN*	5.31	5.79 × 10^−3^	*CST1*	3.1 × 10^−03^	2.66 × 10^−3^
	*HPSE2*	4.54	4.08 × 10^−3^	*SPRR3*	3.3 × 10^−03^	6.00 × 10^−3^
	*NPY5R*	4.51	9.50 × 10^−7^	*KRT13*	4.2 × 10^−03^	3.94 × 10^−4^
	*LINC01833*	4.22	2.95 × 10^−3^	*SPRR1A*	5.3 × 10^−03^	2.05 × 10^−3^
	*SOX8*	4.04	8.42 × 10^−7^	*KRT6C*	5.5 × 10^−03^	6.69 × 10^−4^
	*ASL*	3.88	3.82 × 10^−5^	*SMR3B*	5.9 × 10^−03^	2.82 × 10^−3^
	*PRLR*	3.81	1.99 × 10^−8^	*BPIFA2*	7.1 × 10^−03^	1.67 × 10^−2^

**Table 5:** Elastic Net 49-Gene Classifier.

Symbol	Value	Symbol	Value	Symbol	Value
***A2M***	−0.11	*HMCN1*	−0.065	*PLA2R1*	−0.0434
*ABCA8*	−0.0129	***IPO9***	0.376	***PLAT***	−0.0448
***ACTA2***	−0.1264	*ITPR1*	−0.1121	*PLD1*	−0.0356
*ADAMTS9*	−0.0986	*KRT14*	−0.0359	*PPARGC1A*	−0.0378
*ALDOA*	−0.018	*LDLRAD4*	0.0354	*PRUNE2*	−0.0026
***ANO1***	−0.099	*LGR4*	−0.0391	***RASSF6***	−0.1461
***APOL6***	−0.0977	*LIMA1*	−0.0793	***SEMA3C***	−0.0582
*CARMN*	−0.0091	***LIMCH1***	−0.145	*SH3D19*	−0.0562
*CD9*	−0.0886	*LOC107987158*	0.0363	***SLPI***	−0.1399
*CFH*	−0.0226	*LTF*	−0.0048	*SVIL*	−0.168
*COL17A1*	−0.0151	***MAMLD1***	0.0339	*SYNPO2*	−0.0159
*COL7A1*	−0.0095	***MIR205HG***	−0.0511	*TAGLN*	−0.0321
*COL9A2*	−0.0072	*MLPH*	−0.013	*TNFRSF19*	−0.0054
***DMD***	−0.3178	*MTUS1*	−0.0917	***TP63***	−0.1442
*EFS*	0.0074	*PARP14*	−0.0746	*TPM2*	−0.0175
*EGFR*	−0.0288	*PCYOX1*	−0.1327		
*FRMD4B*	−0.0279	*PIK3R1*	−0.0097		

Note: Genes used in the 14-gene classifier are shown in bold.

**Table 6:** Multivariate Cox Regression Analysis.

Clinical Covariates & Gene Classifier	Values	Hazard Ratio	95% Confidence Interval	*p*-Value
**Vascular Invasion**	No	1		0.097
	Yes	2.703	0.83–8.76	
**Stage**	I–II	1		0.271
	III–IV	1.601	0.69–3.70	
**Gene Classifier**	Group 1	1		0.010
	Group 2	26.01	2.19–309.3	

## Data Availability

RNA sequencing data is available for download from the NCBI BioProject database using study accession number PRJNA287156.

## Supplementary Materials

The following supporting information can be downloaded at: https://www.mdpi.com/article/10.3390/cancers15051390/s1, Figures S1–S6: Larger versions of the heatmaps in Figure 1, Figure 2 and Figure 3, Figure 5, Figure 6 and Figure 8, respectively. Table S1: No Myb group differential gene expression analysis results. Table S2: Poor survival differential gene expression analysis results; File S1: Clinical data analysis details with univariate and bivariate Cox regression results and details of software packages used.
