# Supplementary material for: Dominant Gene Expression Profiles Define Adenoid Cystic Carcinoma (ACC) from Different Tissues: Validation of a Gene Signature Classifier for Poor Survival in Salivary Gland ACC
Source: Cancers (Basel). 2023 Feb 22;15(5):1390. doi: 10.3390/cancers15051390 (PMC10000625; doi:10.3390/cancers15051390)
Supplement: Supplementary file 1 [file cancers-15-01390-s001.zip › FigS1_ACC_Samples_Heatmap.pdf]

Figure S1

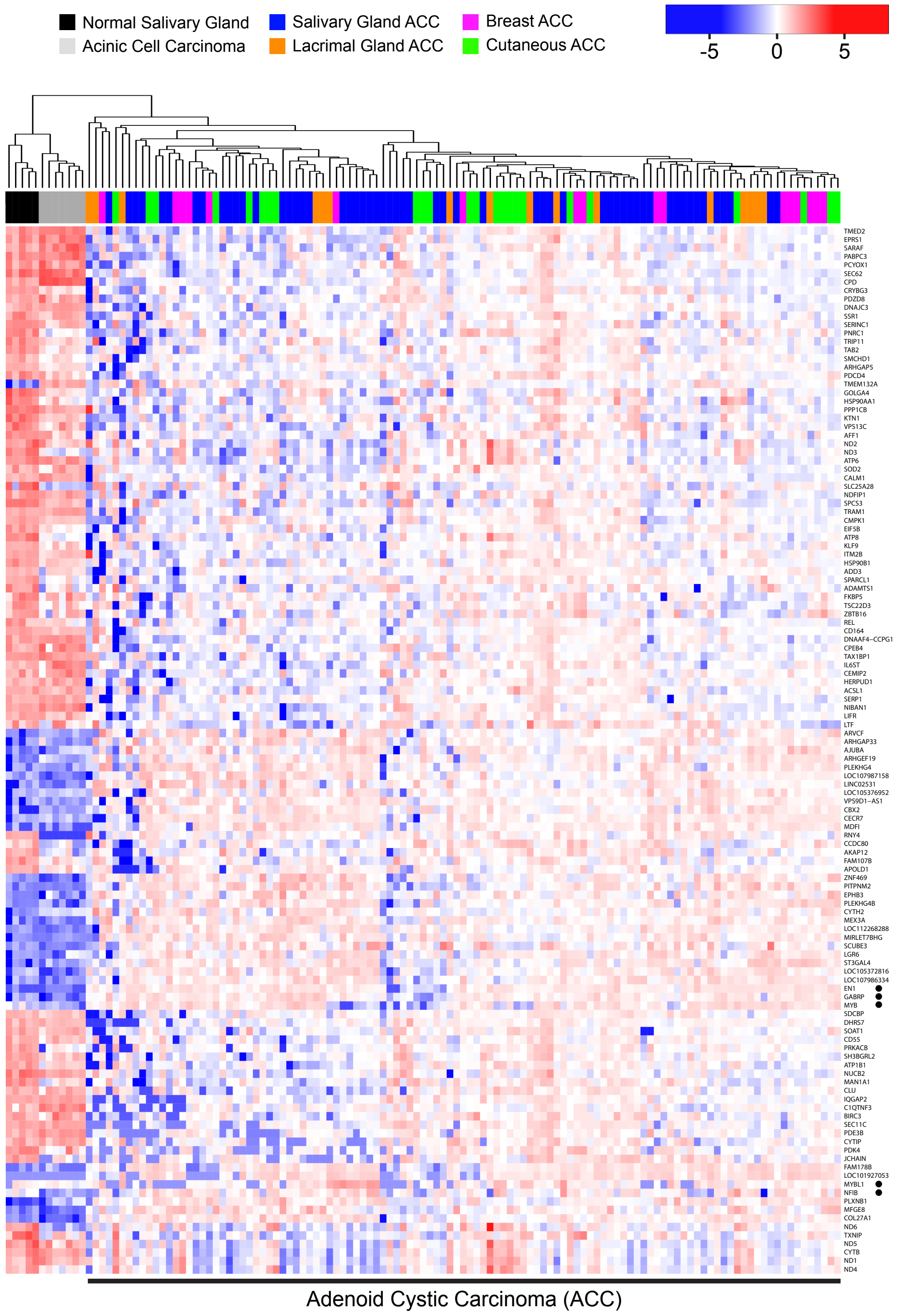

Figure S1. This is a larger version of the heatmap in Figure 1, which summarizes the gene expression differences in ACC samples. The color bar at top identifies the tissue type and tissue of origin. The normal salivary gland and acinic cell carcinoma samples are at left. Several genes important for ACC tumors are marked by dots at right.
