# Supplementary material for: Dominant Gene Expression Profiles Define Adenoid Cystic Carcinoma (ACC) from Different Tissues: Validation of a Gene Signature Classifier for Poor Survival in Salivary Gland ACC
Source: Cancers (Basel). 2023 Feb 22;15(5):1390. doi: 10.3390/cancers15051390 (PMC10000625; doi:10.3390/cancers15051390)
Supplement: Supplementary file 1 [file cancers-15-01390-s001.zip › FigS2_Tissue_Markers.pdf]

Figure S2

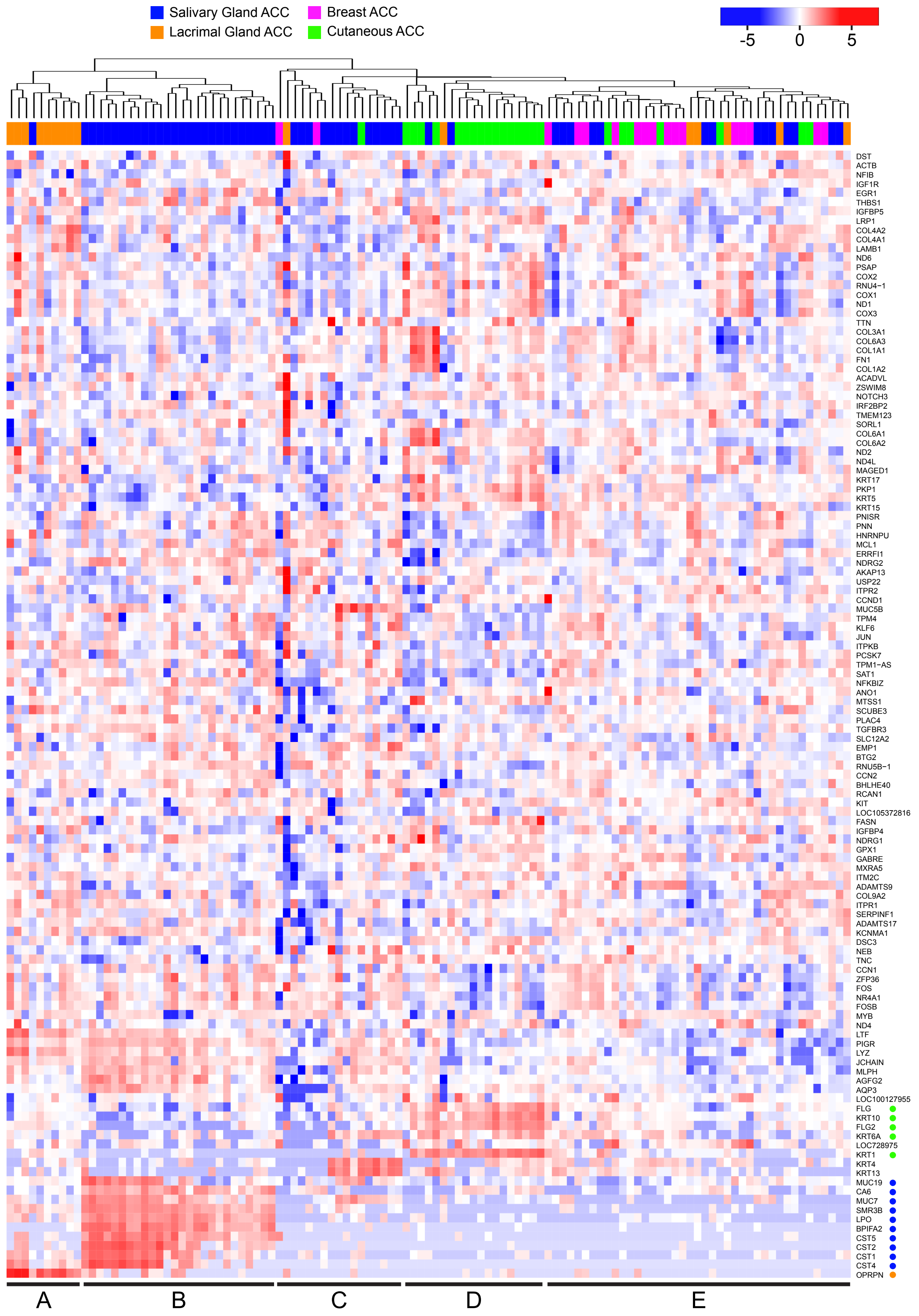

Figure S2. A larger version of the heatmap in Figure 2. Tissue-specific gene expression differenes in ACC tumors. The ACC tumors from the DK cohort were analyzed for tissue-specific gene expression by specifically selecting genes that marked tumors derived from different tissues. A total of 1089 differentially-expressed genes were identified by comparing all the tissue groups to each other (at least 2-fold up- or down-regulated with adjusted p-value < 0.05). The heatmap summarizes the gene expression differences for 123 of the most highly expressed genes. The tissues of origin are indicated in the color bar at top: lacrimal gland, salivary gland, cutaneous and breast are indicated by orange, blue, green and pink, respective-ly. Notable genes mentioned in the text are marked by dots along the right edge.
