# Supplementary material for: Dominant Gene Expression Profiles Define Adenoid Cystic Carcinoma (ACC) from Different Tissues: Validation of a Gene Signature Classifier for Poor Survival in Salivary Gland ACC
Source: Cancers (Basel). 2023 Feb 22;15(5):1390. doi: 10.3390/cancers15051390 (PMC10000625; doi:10.3390/cancers15051390)
Supplement: Supplementary file 1 [file cancers-15-01390-s001.zip › FigS3_Poor_Survival_Heatmap.pdf]

Figure S3

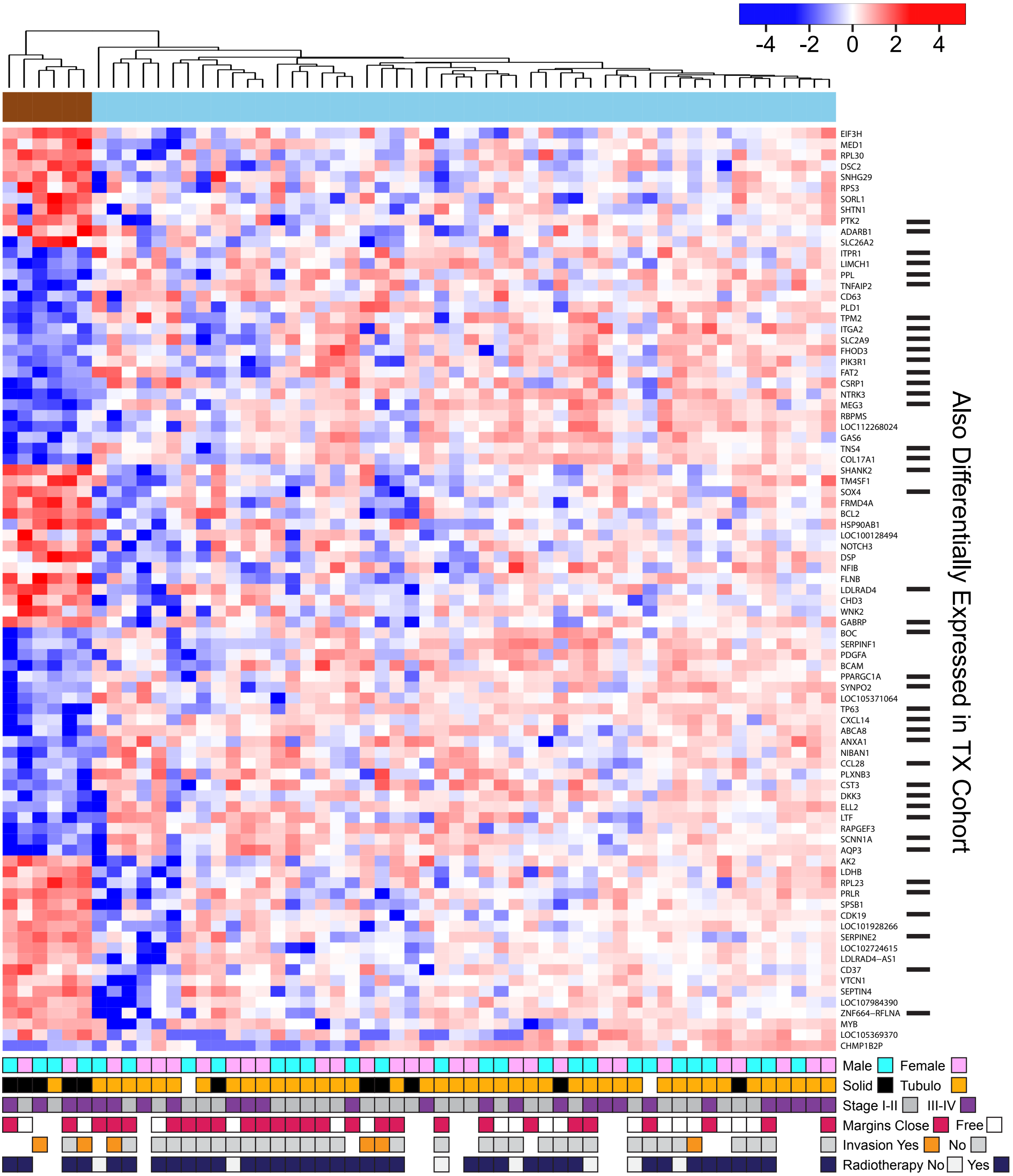

Figure S3. This is a larger version of the heatmap in Figure 3, summarizing the differential gene expression analysis comparing the poor survival (brown) subgroup to the rest of the samples. Genes marked by bars at right were also identified previously in a poor-survival sub-group from the TX cohort [6]. The color bars at the bottom summarize the available clinical information for gender, solid or tubulocribiform morphology, tumor stage, magins, vascular invasion and radiotherapy.
