# Supplementary material for: Dominant Gene Expression Profiles Define Adenoid Cystic Carcinoma (ACC) from Different Tissues: Validation of a Gene Signature Classifier for Poor Survival in Salivary Gland ACC
Source: Cancers (Basel). 2023 Feb 22;15(5):1390. doi: 10.3390/cancers15051390 (PMC10000625; doi:10.3390/cancers15051390)
Supplement: Supplementary file 1 [file cancers-15-01390-s001.zip › FigS4_Heatmap_Combined_Groups.pdf]

Figure S4

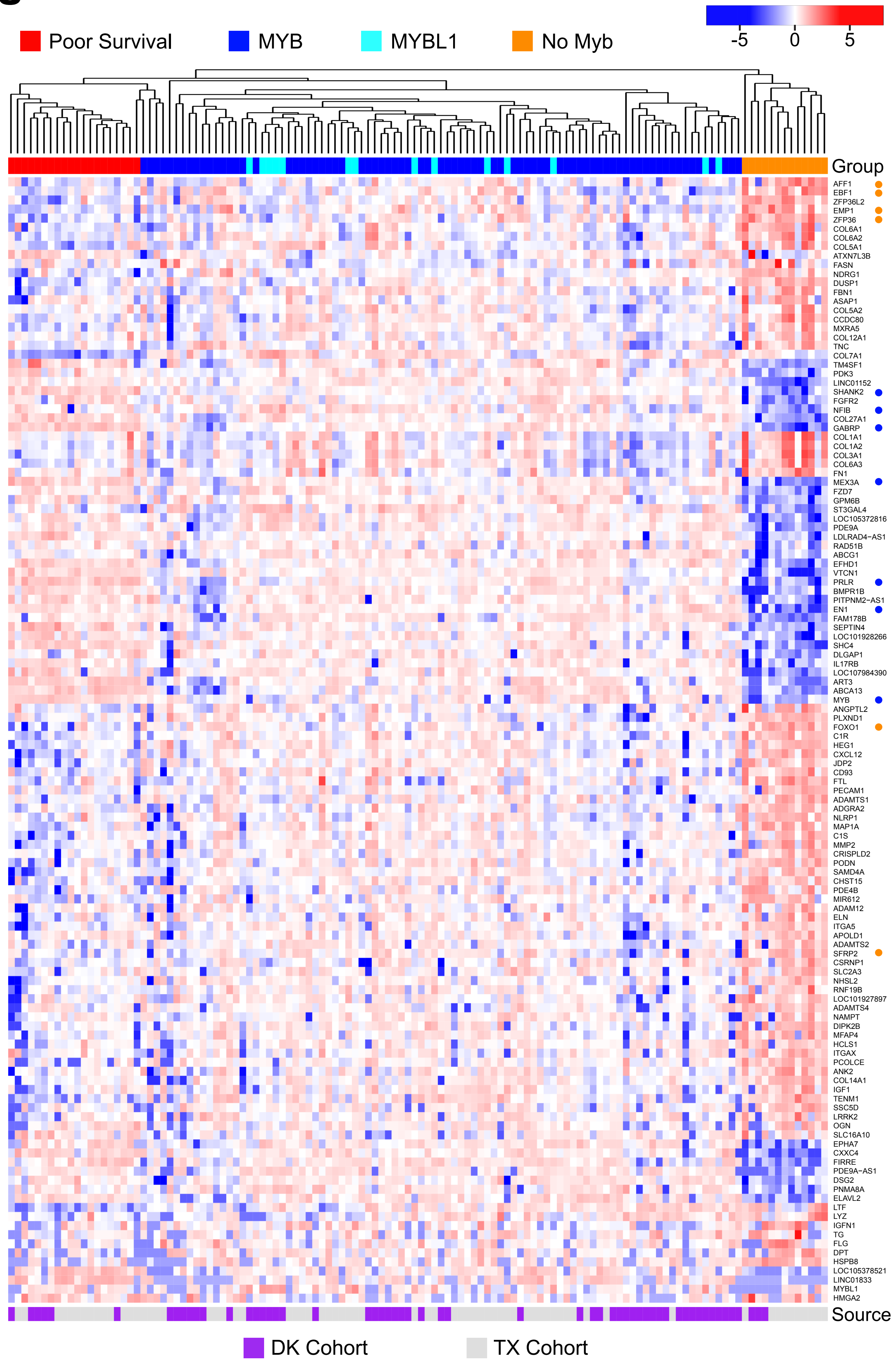

Figure S4. A larger version of the heatmap in Figure 5. Differential gene expression analysis of 'No Myb' samples. The heatmap summarizes the differential gene expression analysis using the combined cohorts of ACC samples from DK and TX, comparing the 'no Myb' group (orange color bar at top) to the rest of the samples, all of which express either MYB or MYBL1. Notable genes mentioned in the text are marked by dots at right. The purple and white color bar at the bottom indicates samples from the DK and TX cohorts, re-spectively.
