# Supplementary material for: Dominant Gene Expression Profiles Define Adenoid Cystic Carcinoma (ACC) from Different Tissues: Validation of a Gene Signature Classifier for Poor Survival in Salivary Gland ACC
Source: Cancers (Basel). 2023 Feb 22;15(5):1390. doi: 10.3390/cancers15051390 (PMC10000625; doi:10.3390/cancers15051390)
Supplement: Supplementary file 1 [file cancers-15-01390-s001.zip › FigS5_Poor_Survival_Heatmap.pdf]

Figure S5

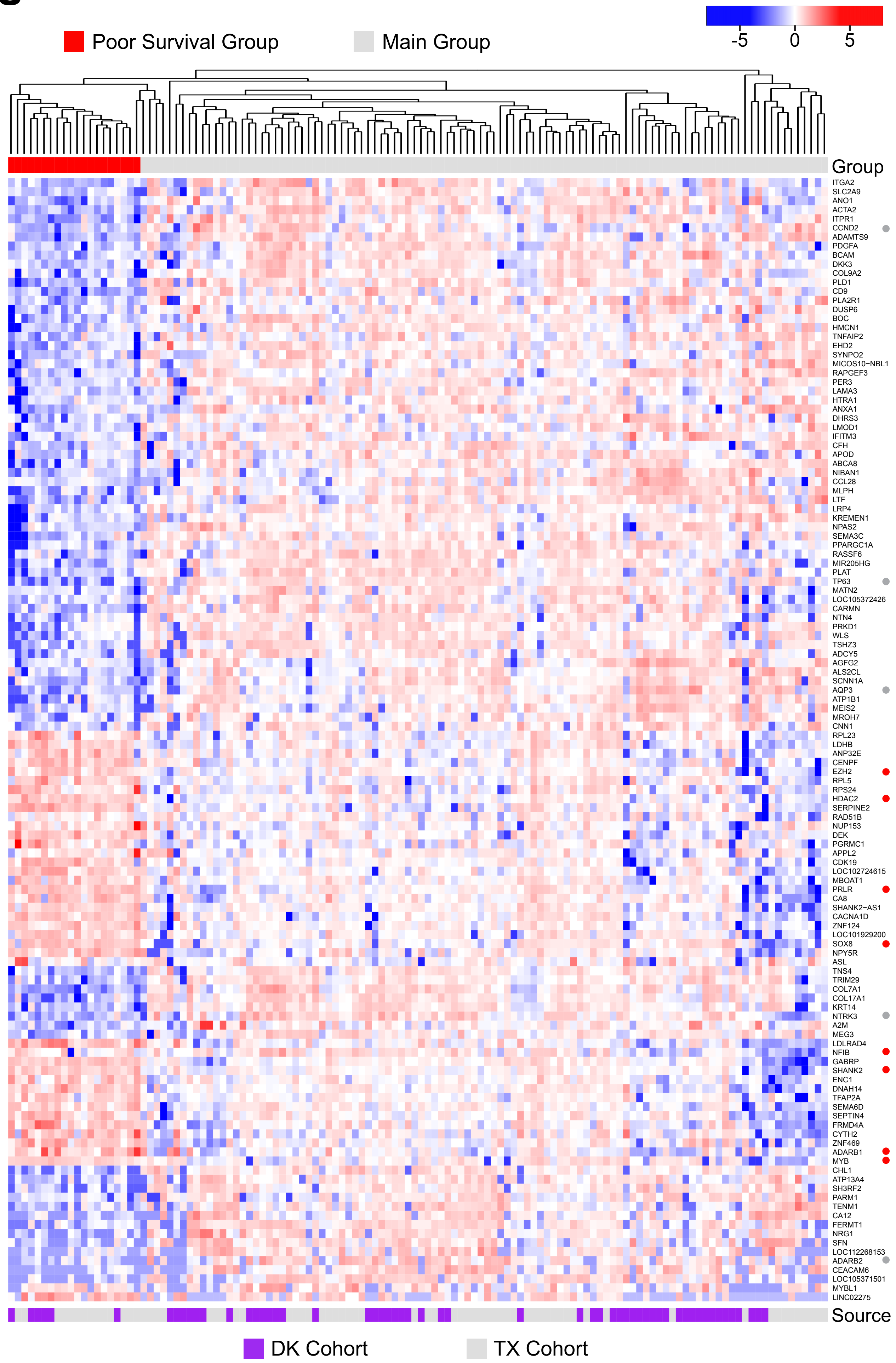

Figure S5. A larger version of the heatmap in Figure 6. Differential gene expression analysis: Poor survival samples. The heatmap summarizes the differential gene expression analysis using the combined cohorts of ACC samples from DK and TX, comparing the poor survival group (red color bar at top) to the rest of the samples. Nota-ble genes mentioned in the text are marked by dots at right. Red and blue dots indicate genes up- or down-regulated in the poor survival samples. The purple and white color bar at the bottom in-dicates samples from the DK and TX cohorts, respectively.
