# Supplementary material for: Dominant Gene Expression Profiles Define Adenoid Cystic Carcinoma (ACC) from Different Tissues: Validation of a Gene Signature Classifier for Poor Survival in Salivary Gland ACC
Source: Cancers (Basel). 2023 Feb 22;15(5):1390. doi: 10.3390/cancers15051390 (PMC10000625; doi:10.3390/cancers15051390)
Supplement: Supplementary file 1 [file cancers-15-01390-s001.zip › FigS6_Classifier_Groups.pdf]

Figure S6

A

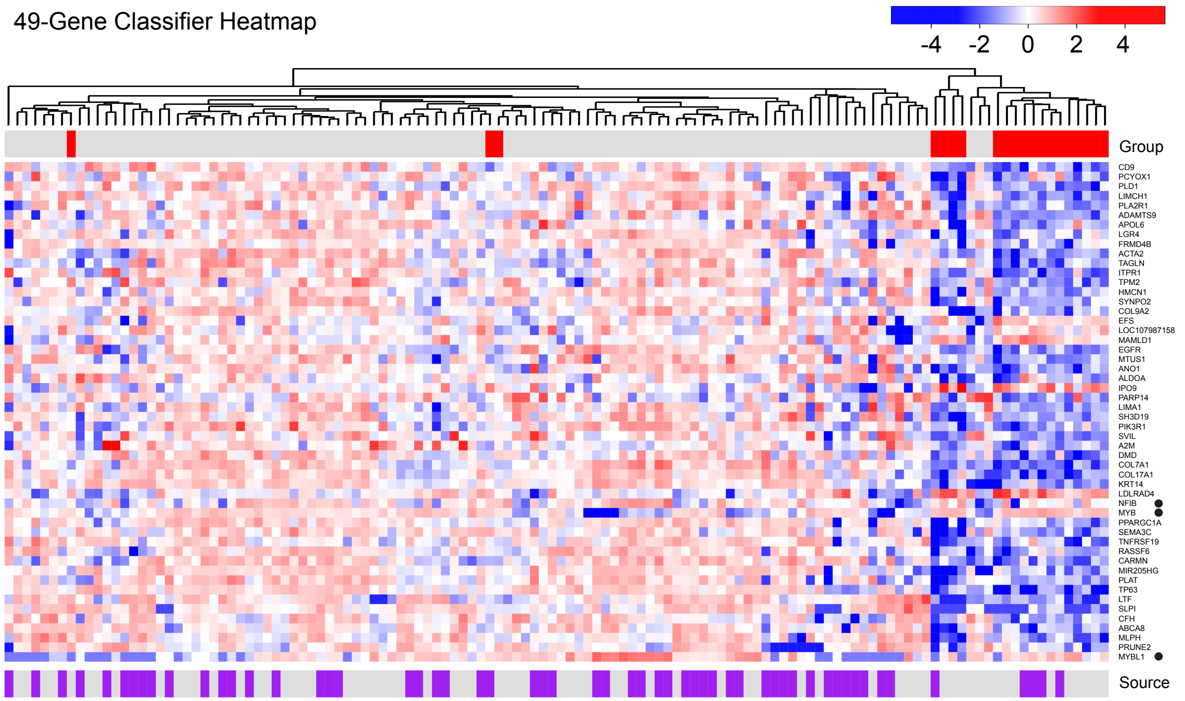

B

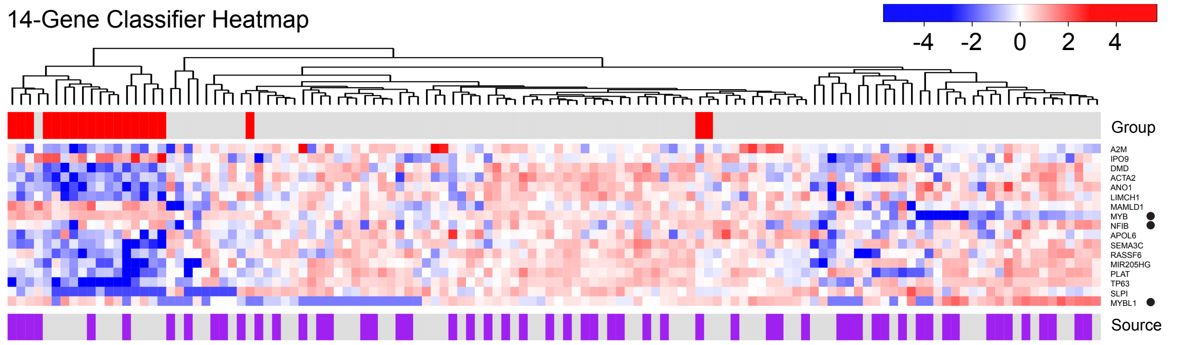

Figure S6. Larger versions of the heatmaps in Figure 8. Classifier Groups. The 49-gene (A) or 14-gene (B) classifiers were used to separate ACC samples into groups by hierarchical clustering, as illustrated in the dendrograms at the top of each heatmap, which compare the gene expression profiles of the two groups. (The MYB, MYBL1 and NFIB genes were added to the analysis for comparison. They are marked by black dots at right.) The samples from the original poor survival group (see Figure 4) are marked by red in the color bars.
