# Supplementary material for: Dominant Gene Expression Profiles Define Adenoid Cystic Carcinoma (ACC) from Different Tissues: Validation of a Gene Signature Classifier for Poor Survival in Salivary Gland ACC
Source: Cancers (Basel). 2023 Feb 22;15(5):1390. doi: 10.3390/cancers15051390 (PMC10000625; doi:10.3390/cancers15051390)
Supplement: Supplementary file 1 [file cancers-15-01390-s001.zip › File S1. Clinal Data Analysis and Tables C1, C2 and C3.pdf]

Clinical Data Analysis

To examine whether the gene classifier provides more information for survival outcomes beyond that contained in the clinical covariates, we performed univariate and multivariate Cox regression analyses with the gene classifier and clinical covariables deemed to be the risk factors as predictors. The available clinical covariables include Margins, Vascular Invasion, Radiotherapy Cribriform and Stage. The analyses were restricted to 56 samples, a union of the subsets to which the data of each variable are available. However, the number of samples used by each Cox regression analysis varies depending on which variables are included due to data availability.

Table C1 shows the results of univariate Cox Regression. We can see that the two variables, Vascular Invasion and Cribriform, are significantly associated with survival outcomes ($p < .05$), while the variable Stage is marginally significant ($p = .084$). We compared these three variables with the gene classifier through bivariate Cox regression (Table C2). The result shows a remarkable association between our gene classifier and survival after adjusting for each clinical covariate's effect. We further performed a multivariate Cox regression (Table C3), and our gene classifier is still significantly correlated with the survival outcomes after adjusting for Vascular Invasion and Stage effects. Note that the Cox regression with three or more variables will not converge if we include Cribriform in the model, which limits our ability to conduct further investigation in this respect. However, the results have given sufficient statistical evidence that our gene classifier provided more information about the survival outcome than the available clinical parameters.

Table C1. Univariate Cox Regression assessing association of individual clinical covariates with survival.

Clinical Covariates & Gene Classifier	Number of Available Samples	Values	Hazard Ratio	95% Confidence Interval	p-value
Margins	44	Close	1		.843
		Free	1.075	.53 – 2.20	
Vascular Invasion	38	No	1		.0048
		Yes	3.989	1.53 – 10.43	
Radiotherapy	45	No	1		.701
		Yes	1.208	.46 – 3.16	
Cribriform	54	Tubular	1		.0208
		Solid	2.368	1.14 – 4.92	
Stage	56	I – II	1		.0839

		III – IV	1.722	.93 – 3.19	
Gene Classifier	56	Group 1	1		.0016
		Group 2	4.694	1.80 – 12.23	

Table C2. Bivariate Cox Regression to compare gene classifier to individual clinical covariates.

Clinical Covariates & Gene Classifier	Variables	Values	Hazard Ratio	95% Confidence Interval	p-value
Vascular Invasion vs. Gene Classifier	Vascular Invasion	No	1		.115
		Yes	2.555	.80 – 8.19	
	Gene Classifier	Group 1	1		.0035
		Group 2	36.382	3.27 – 404.5	
Cribriform vs. Gene Classifier	Cribriform	Tubular	1		.025
		Solid	2.342	1.11 – 4.93	
	Gene Classifier	Group 1	1		.0019
		Group 2	4.748	1.78 – 12.67	
Stage vs. Gene Classifier	Stage	I – II	1		.0426
		III - IV	1.927	1.02 – 3.63	
	Gene Classifier	Group 1	1		.0007
		Group 2	5.629	2.08 – 15.21	

Table C3. Multivariate Cox Regression.

Clinical Covariates & Gene Classifier	Values	Hazard Ratio	95% Confidence Interval	p-value
Vascular Invasion	No	1		.097
	Yes	2.703	.83 – 8.76	
Stage	I – II	1		.271
	III - IV	1.601	.69 – 3.70	
Gene Classifier	Group 1	1		.010
	Group 2	26.01	2.19 – 309.3	

Details of R and Bioconductor Analysis Packages

```
R version 4.1.0 (2021-05-18)
Platform: x86_64-pc-linux-gnu (64-bit)
Running under: Ubuntu 20.04.2 LTS

Matrix products: default
BLAS:   /usr/lib/x86_64-linux-gnu/blas/libblas.so.3.9.0
LAPACK: /usr/lib/x86_64-linux-gnu/lapack/liblapack.so.3.9.0

locale:
 [1] LC_CTYPE=C.UTF-8       LC_NUMERIC=C           LC_TIME=C.UTF-8        LC_COLLATE=C.UTF-8     LC_MONETARY=C.UTF-8
 [6] LC_MESSAGES=C.UTF-8    LC_PAPER=C.UTF-8       LC_NAME=C              LC_ADDRESS=C           LC_TELEPHONE=C
[11] LC_MEASUREMENT=C.UTF-8 LC_IDENTIFICATION=C

attached base packages:
[1] parallel  stats4    stats     graphics  grDevices utils     datasets  methods   base

other attached packages:
 [1] survival_3.2-11                          BSgenome.Hsapiens.NCBI.GRCh38_1.3.1000
 [3] TxDb.Hsapiens.UCSC.hg38.knownGene_3.13.0 GenomicFeatures_1.44.0
 [5] SIFT.Hsapiens.dbSNP137_1.0.0             RSQLite_2.2.7
 [7] org.Hs.eg.db_3.13.0                      rDGIdb_1.18.0
 [9] biomaRt_2.48.0                           topGO_2.44.0
[11] SparseM_1.81                             GO.db_3.13.0
[13] AnnotationDbi_1.54.0                     graph_1.70.0
[15] gage_2.42.0                              pathview_1.32.0
[17] AnnotationHub_3.0.0                      BiocFileCache_2.0.0
[19] dbplyr_2.1.1                             BSgenome_1.60.0
[21] rtracklayer_1.52.0                       VariantAnnotation_1.38.0
[23] Rsamtools_2.8.0                          Biostrings_2.60.1
[25] XVector_0.32.0                           genefilter_1.74.0
[27] DESeq2_1.32.0                            SummarizedExperiment_1.22.0
[29] Biobase_2.52.0                           MatrixGenerics_1.4.0
[31] matrixStats_0.59.0                       GenomicRanges_1.44.0
[33] GenomeInfoDb_1.28.0                      IRanges_2.26.0
[35] S4Vectors_0.30.0                         BiocGenerics_0.38.0
[37] edgeR_3.34.0                             limma_3.48.0
[39] gtools_3.9.2                             lattice_0.20-44
[41] diagram_1.6.5                            shape_1.4.6
[43] xtable_1.8-4                             rgl_0.106.8
[45] plot3D_1.4                               ggplot2_3.3.3
[47] gplots_3.1.1                             RColorBrewer_1.1-2
[49] checkmate_2.0.0                          R.utils_2.10.1
[51] R.oo_1.24.0                              R.methodsS3_1.8.1

loaded via a namespace (and not attached):
 [1] backports_1.2.1        splines_4.1.0          BiocParallel_1.26.0
 [4] crosstalk_1.1.1        digest_0.6.27          htmltools_0.5.1.1
 [7] fansi_0.5.0            magrittr_2.0.1         memoise_2.0.0
[10] annotate_1.70.0        prettyunits_1.1.1      colorspace_2.0-1
[13] blob_1.2.1             rappdirs_0.3.3         xfun_0.23
[16] dplyr_1.0.6            tcltk_4.1.0            crayon_1.4.1
[19] RCurl_1.98-1.3         jsonlite_1.7.2         glue_1.4.2
[22] gtable_0.3.0           zlibbioc_1.38.0        DelayedArray_0.18.0
[25] Rgraphviz_2.36.0       scales_1.1.1           DBI_1.1.1
[28] miniUI_0.1.1.1         Rcpp_1.0.6             progress_1.2.2
[31] bit_4.0.4              htmlwidgets_1.5.3      httr_1.4.2
[34] ellipsis_0.3.2         pkgconfig_2.0.3        XML_3.99-0.6
[37] locfit_1.5-9.4         utf8_1.2.1             tidyselect_1.1.1
```

```
[40] rlang_0.4.11                manipulateWidget_0.11.0   later_1.2.0
[43] munsell_0.5.0               BiocVersion_3.13.1        tools_4.1.0
[46] cachem_1.0.5                generics_0.1.0            evaluate_0.14
[49] stringr_1.4.0               fastmap_1.1.0             yaml_2.2.1
[52] knitr_1.33                  bit64_4.0.5               caTools_1.18.2
[55] purrr_0.3.4                 KEGGREST_1.32.0           mime_0.10
[58] KEGGgraph_1.52.0            compiler_4.1.0            rstudioapi_0.13
[61] filelock_1.0.2              curl_4.3.1                png_0.1-7
[64] interactiveDisplayBase_1.30.0 tibble_3.1.2            geneplotter_1.70.0
[67] stringi_1.6.2               Matrix_1.3-4              vctrs_0.3.8
[70] pillar_1.6.1                lifecycle_1.0.0           BiocManager_1.30.15
[73] bitops_1.0-7                httpuv_1.6.1              R6_2.5.0
[76] BiocIO_1.2.0                promises_1.2.0.1          KernSmooth_2.23-20
[79] assertthat_0.2.1            rjson_0.2.20              withr_2.4.2
[82] GenomicAlignments_1.28.0    GenomeInfoDbData_1.2.6    hms_1.1.0
[85] grid_4.1.0                  rmarkdown_2.8             misc3d_0.9-0
[88] shiny_1.6.0                 restfulr_0.0.13
```
